# A Phase II Trial of the Double Epigenetic Priming Regimen Including Chidamide and Decitabine for Relapsed/Refractory Acute Myeloid Leukemia

**DOI:** 10.3389/fonc.2021.726926

**Published:** 2021-09-03

**Authors:** Jia Yin, Chao-Ling Wan, Ling Zhang, Hao Zhang, Lian Bai, Hai-Xia Zhou, Ming-Zhu Xu, Li-Yun Chen, Chong-Sheng Qian, Hui-Ying Qiu, Su-Ning Chen, Xiao-Wen Tang, De-Pei Wu, Yan-Ming Zhang, Ai-Ning Sun, Sheng-Li Xue

**Affiliations:** ^1^National Clinical Research Center for Hematologic Diseases, Jiangsu Institute of Hematology, The First Affiliated Hospital of Soochow University, Suzhou, China; ^2^Institute of Blood and Marrow Transplantation, Collaborative Innovation Center of Hematology, Soochow University, Suzhou, China; ^3^Department of Hematology, The Affiliated Hospital of Jining Medical College, Jining, China; ^4^Department of Hematology, Canglang Hospital of Suzhou, Suzhou, China; ^5^Department of Hematology, The Affiliated Huai’an Hospital of Xuzhou Medical University and The Second People’s Hospital of Huai’an, Huai’an, China

**Keywords:** epigenomics, histone deacetylase inhibitor (HDACi), CDIAG regimen, relapsed/refractory acute myeloid leukemia, salvage therapy

## Abstract

**Objective:**

To explore the role of chidamide, decitabine plus priming regimen in the salvage treatment of relapsed/refractory acute myeloid leukemia.

**Methods:**

A clinical trial was conducted in relapsed/refractory acute myeloid leukemia patients using chidamide, decitabine, cytarabine, idarubicin, and granulocyte-colony stimulating factor, termed CDIAG, a double epigenetic priming regimen.

**Results:**

Thirty-five patients were recruited. Three patients received 2 treatment cycles. In 32 evaluable patients and 35 treatment courses, the completed remission rate (CRR) was 42.9%. The median OS time was 11.7 months. The median OS times of responders were 18.4 months, while those of nonresponders were 7.4 months (P = 0.015). The presence of RUNX1 mutations was associated with a high CRR but a short 2-year OS (P = 0.023) and PFS (P = 0.018) due to relapse after treatment. The presence of IDH mutations had no effect on the remission rate (80.0% *vs*. 73.3%), but showed a better OS (2-year OS rate: 100.0% *vs*. 28.9%). Grade 3/4 nonhematological adverse events included pneumonia, hematosepsis, febrile neutropenia, skin and soft tissue infection and others.

**Conclusion:**

The double epigenetic priming regimen (CDIAG regimen) showed considerably good antileukemia activity in these patients. Adverse events were acceptable according to previous experience. The study was registered as a clinical trial.

**Clinical Trial Registration:**

https://clinicaltrials.gov/, identifier:NCT03985007

## Introduction

Although treatment of Acute myeloid leukemia (AML) is rapidly progressing, approximately 10% to 40% of newly diagnosed AML patients cannot achieve complete remission (CR) through induction chemotherapy, and more than 50% of AML patients will ultimately relapse (1). For patients with relapsed/refractory (R/R) AML, the goal of chemotherapy varies from achieving long-term remission to providing a “bridge” to stem cell transplantation (SCT). Most conventional chemotherapeutic drugs have a low reinduction remission rate of nearly 1/3, poor tolerability and a prolonged bone marrow (BM) suppression stage, often leading to serious infection, high mortality, and a short survival (2). Therefore, it is crucial to explore and formulate reasonable and effective combined therapeutic strategies to undergo curative treatment with allogeneic stem cell transplantation (allo-SCT) in CR status (3).

Although several new small-molecule inhibitors have been developed (e.g., ABT-199, midostaurin, and *IDH1/2* inhibitor) and have shown promising results in R/R AML treatment, they are not currently commercially available in mainland China. In recent decades, epigenetic treatment for hypermethylation or histone deacetylation has been a major breakthrough in AML treatment (4). The application of DNA demethylation drugs involved in epigenetic regulation to elderly (age ≥ 60 years) AML and R/R AML patients was the IA category recommendation for first-line induction therapy in the NCCN guidelines (5). Chidamide is the first subtype-selective oral histone deacetylation inhibitor (HDACi) commercially available in mainland China and has been certified internationally by the FDA because it is effective in treating R/R peripheral T-cell lymphoma (PTCL) (6). Chidamide possesses potent HDAC inhibitory properties by terminating the deacetylation of histones H3 and H4 *via* inhibiting HDAC types 1, 2, 3, and 10. Selective targeting of individual HDACs causes differentiation, apoptosis, cell cycle inhibition, migration inhibition, susceptibility to chemotherapy and anti-angiogenesis (7, 8).

In the treatment of R/R AML with low-dose cytarabine and anthracycline combined with granulocyte-colony stimulating factor (G-CSF) (priming regimen) (9), the sensitizing effect of hematopoietic growth factors on leukemic cells enhances the cytotoxicity of chemotherapy in AML. Previous studies have suggested that the combination of decitabine with G-CSF, low-dose cytarabine and aclarubicin (DCAG) improved the CR rate and was well-tolerated in newly diagnosed elderly AML patients (10). Moreover, patients with R/R or high-risk AML were treated with the DCAG regimen, which was proven to overcome drug resistance and improve therapeutic efficacy (11). HDACis in monotherapy are modestly active in high-risk myelodysplastic syndrome (MDS) and AML, and *in vitro* evidence supports the synergy between hypomethylating agents (HMAs) and HDACis (12). Decitabine used concurrently or sequentially with vorinostat (an HDACi) was safe and well tolerated in patients with R/R AML (n=29), with responses observed in 15% of patients (13). Several of the above rationales led us to design a regimen that included chidamide, decitabine, idarubicin, cytarabine, and G-CSF (the CDIAG double epigenetic priming regimen) to treat patients with R/R AML.

## Materials and Methods

### Patients

The trial was conducted at four medical centers (the First Affiliated Hospitals of Soochow University, Affiliated Hospital of Jining Medical University, Second People’s Hospital of Huai’an, and Canglang Hospital of Suzhou), and the investigational agent chidamide was provided by Shenzhen Chipscreen Biosciences Ltd. (Shenzhen, China) under an agreement. All study subjects provided their voluntary, written informed consent. The current study was conducted in accordance with the Declaration of Helsinki. The protocol and all its amendments were approved by the Ethics Committee of the First Affiliated Hospital of Soochow University (ClinicalTrials.gov identifier NCT03985007).

**Figure 1:**
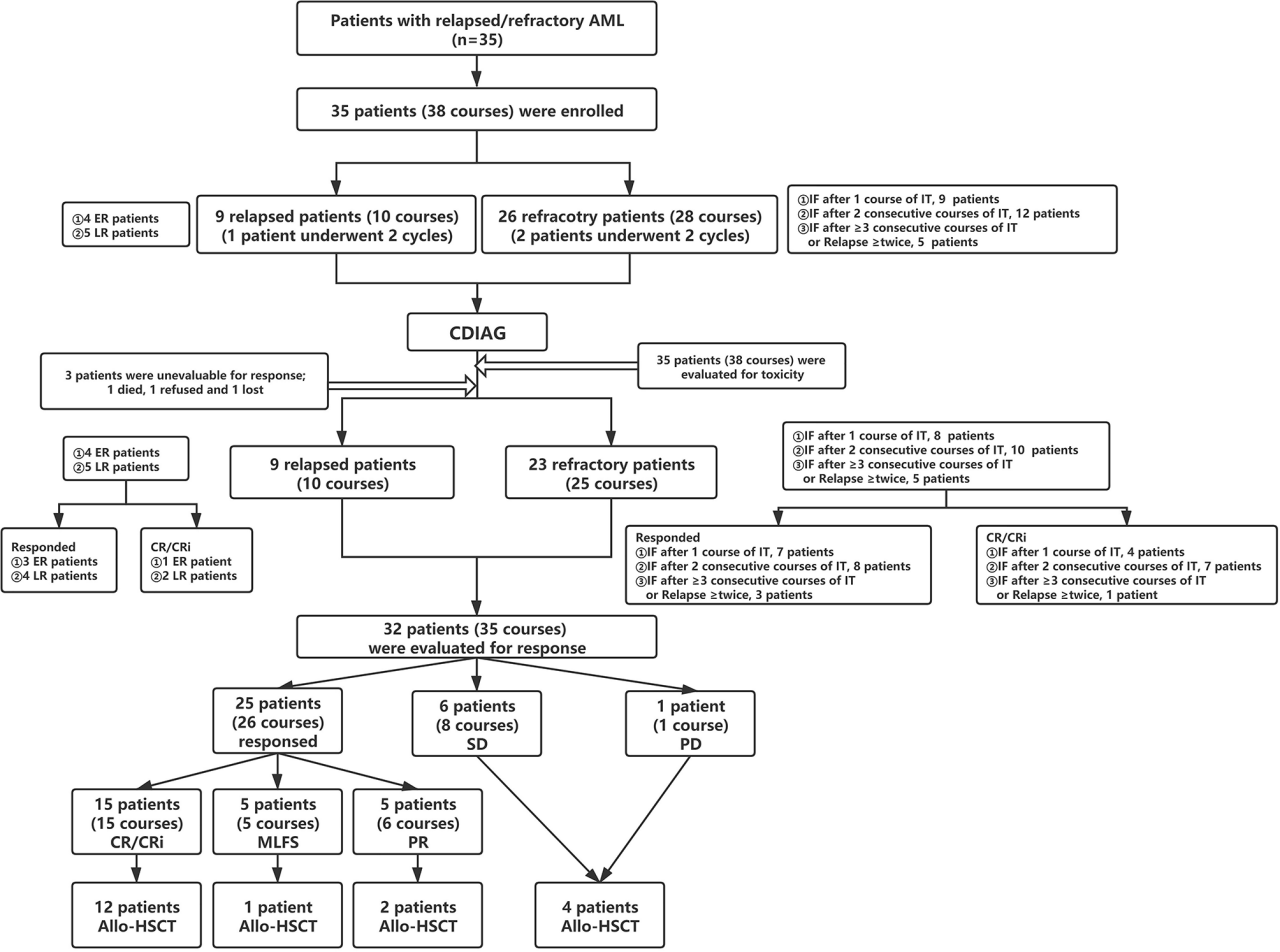
Flow diagram for patient categorization and treatment. AML, acute myeloid leukemia; ER, early relapse; LR, late relapse; IT, induction therapy; IF, induction failure; CR, complete remission; Cri, CR with incomplete hematologic recovery; MLFS, morphologic leukemia-free state; PR, partial response; SD, stable disease, PD, progressive disease; Allo-HSCT, allogeneic hematopoietic stem cell transplantation.

Eligible patients met the R/R AML [non-acute promyelocytic leukemia (non-APL)] criteria (**Figure 1** and **Supplemental Table 1**). At enrollment, the patients were required to be 18 to 70 years of age and have an Eastern Cooperative Oncology Group (ECOG) performance status score less than 3, adequate organic function, and no severe complications, such as active infections and bleeding. Women of childbearing potential were required to practice adequate birth control while participating in the protocol. The exclusion criteria were as follows: unable to tolerate induction chemotherapy and a life expectancy of less than 1 month. The principal investigators performed BM morphology, immunophenotyping, cytogenetics, and molecular genetic analyses by reviewing central laboratory reports.

### Study Design and Objectives

The therapeutic regimen comprised chidamide (30 mg orally twice every week for 2 weeks on days 1, 4, 8, and 11), decitabine [20 mg/m^2^ intravenously daily for 5 days (d1-d5)], and the IAG regimen [cytarabine (10 mg/m^2^ subcutaneously every 12 hrs. on days 4-17), idarubicin (5 mg intravenously every other day on days 4, 6, 8, 10, 12, and 14), and concurrent G-CSF (200 μg/m^2^/day subcutaneously daily on days 3-17)] (**Supplemental Table 2**). The patients were removed from the study therapy for disease progression, symptomatic deterioration, or per patient request. Subsequent therapy after CDIAG for patients who did not receive SCT is described in detail in **Supplemental Table 3**. Supportive treatments, including G-CSF, the transfusion of RBCs or PLTs, and antibiotics, could be routinely administered during CDIAG treatment.

The primary objective of this phase II trial was to evaluate the ORR (confirmed CR, CRi, MLFS and PR) and CR (confirmed CR and CRi) rate by a BM examination based on central site review (**Supplemental Table 4**). The secondary objectives were to estimate the OS, PFS, and RFS and to assess toxicity. The OS duration was measured from the onset of CDIAG treatment until death due to any cause or censored for patients who remained alive at the time of assessment. PFS was defined as the time from the date of entry into the trial until the date of disease progression at any site, including distant metastasis or second primary tumors, or death. RFS was defined only for patients who achieved CR or CRi and was measured from the date of achieving remission until the date of relapse or death from any cause. Patients not known to have relapsed or died at the last follow-up were censored on the date they were last examined.

### Assessments

Clinical data, biologic data (BM smears and MRD with 10-color MFC afforded 1:10^-4^ to 1:10^-5^ level sensitivity]), and response assessment were centrally reviewed. Twenty-four days after the start of treatment (7 days after the end of therapy), the efficacy was evaluated in the BM. Patients who did not achieve CR/CRi received a BM examination again 31 days after the commencement of treatment (14 days after the end of therapy), and the best BM response was documented. Patients who did not achieve CR/CRi after both assessments using our salvage chemotherapy regimen were allowed to receive a second course, but the evaluation was conducted for each course. Routine blood counts were monitored every day, and electrolyte levels, liver function, and creatinine levels were monitored twice weekly following CDIAG chemotherapy.

The response conditions were defined according to the 2017 ELN recommendations (3). Investigator-assessed AEs were graded according to the National Cancer Institute’s Common Terminology Criteria for Adverse Events (NCI CTCAE version 5.0). Treatment-related mortality (TRM), adverse reactions in hematology (agranulocytosis days, PLT/RBC transfusion units) and nonhematological adverse reactions (**Supplemental Table 5**) (infection and organ injury) were recorded to evaluate toxicities. TRM was defined as death within 28 days after the initiation of IT.

### Statistical Analysis

Thirty-five eligible patients were enrolled in this study. Standard statistical methods were used for all analyses in the trial: T-test for means between two groups, single-factor and multi-level variance analyses for multiple groups, Fisher’s exact test for categorical endpoints, Kaplan-Meier curves and the log-rank test for the time-to-event endpoints. Descriptive statistics (counts and percentages for categorical variables; mean and standard deviation, and medians and range for continuous variables) were used throughout the study. *P* values of 0.05 were considered significant for analysis. All statistical analyses were performed with Graphpad Prism (version 8.0.2). Patient age, sex, WHO classification, WBC count, BM blasts, SCT, previous HMA exposure (before CDIAG regimen), prognosis risk, response and R/R status, as well as treatments before CDIAG, were examined to assess their impact on the survival and remission rates. The follow-up cutoff date was defined as the end of June 2020.

## Results

### Patient Characteristics

Thirty-five patients from four institutions who met the eligibility criteria were registered between 12/15/2016 and 03/29/2020 (**Table 1**). There were 19 male and 16 female patients, with a median age of 39.5 years (range, 18 to 68 years). The 35 patients included 28 (28/35, 80.0%) patients with AML, not otherwise specified (AML, NOS), 5 (5/35, 14.3%) patients with AML with myelodysplasia-related changes (AML-MRC), 1 (1/35, 2.9%) patient with AML with t(8;21)(q22;q22.1)/*RUNX1*-*RUNX1T1* and 1 (1/35, 2.9%) patient with AML with inv (16)(p13.1q22)/*CBFβ-MYH11*(concurrent with a *KIT* mutation). The most frequently mutated genes were *FLT3*-ITD (25.7%), *DNMT3A* (25.7%), *NPM1* (20.0%), *CEBPα* (20.0%), *WT1* (20.0%), *TET2* (17.1%), *IDH1/2* (14.3%), *NRAS* (11.4%) and *RUNX1* (11.4%).

**Table 1:** Characteristics of the 35 enrolled patients.

Characteristic	Value
Relapsed/refractory	9/26
Male/female, No.	19/16
Age, median (range), y	39.5 (18-68)
WBC count, median (range), ×10exp9/L	26.0 (1.0-299.0)
Hemoglobin level, median (range), g/L	76 (48-127)
Platelet count, median (range), ×10exp9/L	54 (10-376)
BM blasts, median (range), %	63.0 (10-97.5)
WHO classification, No. (%)	
AML, NOS	28 (80.0)
AML with MRC	5 (14.3)
AML with t(8; 21)	1 (2.9)
AML with inv(16)	1 (2.9)
Prognosis risk for R/R AML, No. (%)	
Favorable	2 (5.7)
Intermediate	4 (11.4)
Poor	29 (82.9)
Prior HMA exposure (before the CDIAG regimen), No. (%)	
0	27 (77.1)
≥ 1	8 (22.9)
Subgroup classification of R/R AML, No. (%)	
Early relapse	4 (11.4)
Late relapse	5 (14.3)
IF after 1 course of IT	9 (25.7)
IF after 2 consecutive courses of IT	12 (34.3)
IF after ≥ 3 consecutive courses of IT	3 (8.6)
Relapse ≥ twice	2 (5.7)
Therapy after regimen, No. (%)	
SCT	19 (54.3)
Others	16 (45.7)
Genes Mutated, No. (%)	
*FLT3*-ITD mutated	9 (25.7)
*DNMT3A* mutated	9 (25.7)
*NPM1* type A mutated	7 (20.0)
*CEBPα* biallelic mutated	7 (20.0)
*WT1* mutated	7 (20.0)
*TET2* mutated	6 (17.1)
*IDH1/IDH2* mutated	5 (14.3)
*RUNX1* mutated	4 (11.4)
*NRAS* mutated	4 (11.4)
*FLT3-TKD* mutated	3 (8.6)
*U2AF1* mutated	2 (5.7)
*TP53* mutated	2 (5.7)

R/R AML, relapsed/refractory AML; AML, NOS, AML, not otherwise specified; AML with MRC, AML with myelodysplasia-related changes; HMA, hypomethylating agent; SCT, stem cell transplantation; WBC, white blood cell; FLT3, FMS-like tyrosine kinase 3, FLT3-ITD, FLT3-internal tandem duplication; DNMT3A, DNA-methyltransferase 3A; NPM1, nucleophosmin 1; CEBPα, CCAAT/enhancer binding protein alpha; WT1, Wilms’ tumor 1; IDH1, isocitrate dehydrogenase 1; IDH2, isocitrate dehydrogenase 2; TET2, Tet methylcytosine dioxygenase 2; RUNX1, runt-related transcription factor 1; NRAS, neuroblastoma RAS viral oncogene homolog; TP53, tumor protein 53; FLT3-TKD, FLT3-tyrosine kinase domain; U2AF1, U2 small nuclear RNA auxiliary factor 1.

Among the 35 patients, three were not evaluable for response and were refractory. Three of the remaining 32 eligible patients had completed 2 cycles; therefore, 32 patients and 35 courses were examined to assess efficacy (**Supplemental Table 6**). Regarding the disease status before CDIAG, 23 patients (25 courses) were refractory, and 9 patients (10 courses) relapsed. Four patients relapsed within 6 months (early relapse), 5 relapsed beyond 6 months (late relapse), 8 experienced induction failure (IF) after 1 course of IT (induction therapy), 10 had IF after 2 consecutive courses of IT, 3 had IF after ≥ 3 consecutive courses of IT, and 2 relapsed more than twice. On registering for this study, 2 patients were categorized as favorable risk, 4 as intermediate risk, and 26 as adverse risk with a poor prognosis according to the prognostic scoring system of R/R AML (**Supplemental Table 7**) (14). Nineteen of 32 (59.4%) eligible patients received allo-SCT after undergoing the prior CDIAG regimen (3 sibling donor type, 1 unrelated donor type, and 15 haploidentical donor type). Seven of 32 (21.9%) evaluable patients had received more than one cycle of HMA therapy before CDIAG.

### Outcomes

**Figure 2:**
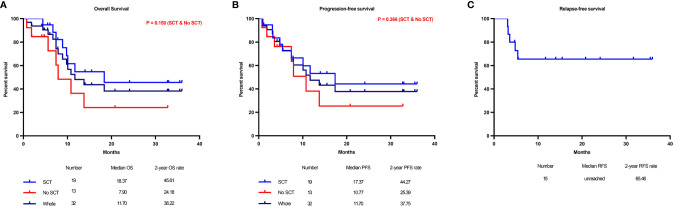
Main study results. Kaplan–Meier graphs illustrating the overall survival **(A)** and progression-free survival **(B)** of all 32 refractory/relapsed (R/R) acute myeloid leukemia (AML) patients after the CDIAG regimen and the distinction of overall survival between patients with or without transplantation. The 2-year relapse-free survival (RFS) rate was 40.7% in 15 patients who achieved CR/CRi **(C)**.

Among the 35 patients, three withdrew before the evaluation. The overall response rate (ORR) for 35 assessable courses in 32 patients was 74.3% (95% confidence interval (CI): 59%-86%), the CR/CR with incomplete hematologic recovery (CRi) rate was 42.9% (95% CI: 25.6%-60.1%), the morphologic leukemia-free state (MLFS) rate was 14.3% (n=5), and the partial remission (PR) rate was 17.1% (n= 6). The stable disease (SD) rate was 22.9% (n= 8), and the progressive disease (PD) rate was 2.9% (n= 1). The median follow-up time was 22.1 months (range, 8.2-48.6 months) for this patient cohort. The median overall survival (OS) time was 11.7 months, and the median progression-free survival (PFS) time was 11.7 months. The survival outcomes of the entire cohort of 32 patients are shown in **Figure 2**. The 2-year OS, PFS and relapse-free survival (RFS) rates were 38.2% (**Figure 2A**), 37.8% (**Figure 2B**), and 65.5% (**Figure 2C**), respectively (RFS was evaluated in 15 patients who achieved CR/CRi). The primary and secondary endpoints are summarized in **Table 2**.

**Table 2:** Primary and secondary patient endpoints.

Endpoint (evaluable patients=32, courses= 35)	Value
Overall response, No. (%)	26 (74.3%)
Complete remission, No. (%)	15 (42.9%)
CR, No. (%)	9 (25.7%)
CRi, No. (%)	6 (17.1%)
Morphologic leukemia-free state, No. (%)	5 (14.3%)
Partial remission, No. (%)	6 (17.1%)
Stable disease, No. (%)	8 (22.9%)
Progressive disease, No. (%)	1 (2.9%)
Median OS time	11.7 months
Median PFS time	11.7 months
2-year OS rate	38.2%
2-year PFS rate	37.8%
2-year RFS rate	65.5%

OS, overall survival; PFS, progression-free survival; RFS, relapse-free survival.

The ORR and CRR were evaluated for 35 courses in 32 patients. OS and PFS were evaluated in 32 patients. RFS was evaluated in 15 patients who achieved CR.

The ORR for the 10 CDIAG induction courses in 9 relapsed patients was 70.0% (7/10) (3 of 4 courses in 4 patients who had an early relapse and 4 of 6 courses in 5 patients who had a late relapse before CDIAG). The CR/CRi rate for the 10 courses in 9 relapsed patients was 30.0% (3/10) (only 1 of 4 courses in 4 patients who had an early relapse and 2 of 6 courses in 5 patients who had a late relapse). No difference was found in the ORR/CRR or survival rates between patients who had early and late relapses (**Table 3**).

**Table 3:** Clinical responses of R/R AML patients with subgroup univariate analysis.

Variable	Response	P-Value	OR* (95% CI)	CR/CRi	P-Value	OR* (95% CI)
Overall	26/35 (74.3%)			15/35 (42.9%)		
Age						
<51 years	22/28 (75.6%)	0.340	0.364 (0.063-2.089)	13/28 (46.4%)	0.669	0.462 (0.076-2.793)
≥51 years	4/7 (57.1%)			2/7 (28.6%)		
Sex						
Male	16/21 (76.2%)	1.000	1.280 (0.276-5.934)	11/21 (52.4%)	0.163	2.750 (0.651-11.624)
Female	10/14 (71.4%)			4/14 (28.6%)		
Blast						
<0.3	3/8 (37.5%)	**0.015**	9.583 (1.613-56.952)	2/8 (25.0%)	**0.450**	2.786 (0.475-16.345)
≥0.3	23/27 (85.2%)			13/27 (48.1%)		
WBC						
<14 × 10E9/L	7/12 (58.3%)	0.220	3.393 (0.703-16.385)	3/12 (25.0%)	0.123	3.273 (0.700-15.291)
≥14 × 10E9/L	19/23 (82.6%)			12/23 (52.2%)		
HB						
<65 g/L	7/11 (63.6%)	0.416	2.171 (0.450-10.486)	2/11 (18.2%)	0.069	5.318 (0.943-29.993)
≥65 g/L	19/24 (79.2%)			13/24 (54.2%)		
PLT						
<40 × 10E9/L	12/14 (85.7%)	0.262	0.333 (0.058-1.919)	7/14 (50.0%)	0.486	0.615 (0.157-2.419)
≥40 × 10E9/L	14/21 (66.7%)			8/21 (38.1%)		
Previous HMA or not						
Yes	5/7 (71.4%)	0.632	0.625 (0.093-4.222)	3/7 (42.9%)	0.576	0.813 (0.150-0.404)
No	20/25 (80.0%)			12/25 (48.0%)		
Prognostic score of R/R AML*						
Favorable/intermediate risk	5/7 (71.4%)	1.000	1.200 (0.189-7.628)	2/7 (28.6%)	0.672	2.167 (0.358-13.110)
Adverse risk	21/28 (75.0%)			13/28 (46.4%)		
Relapsed/Refractory						
Relapsed	7/10 (70.0%)	0.694	1.357 (0.265-6.958)	3/10 (30.0%)	0.458	2.154 (0.451-10.287)
Refractory	19/25 (76.0%)			12/25 (48.0%)		
Relapsed/Refractory subgroup						
Early relapse	3/4 (75.0%)	0.765	0.909 (0.484-1.705)	1/4 (25.0%)	0.661	1.132 (0.651-1.969)
Late relapse	4/6 (66.7%)			2/6 (30.0%)		
IF after 1 course of IT	7/8 (87.5%)			4/8 (50.0%)		
IF after 2 consecutive courses of IT	9/12 (75.0%)			7/12 (58.3%)		
IF after ≥ 3 consecutive courses of IT or relapse ≥ twice	3/5 (60.0%)			1/5 (20.0%)		
Genes Mutated						
*FLT3*-ITD^mut^	7/10 (70.0%)	0.694	0.737 (0.144-3.778)	3/10 (30.0%)	0.458	0.464 (0.097-2.217)
*FLT3*-ITD^wt^	19/25 (76.0%)			12/25 (48.0%)		
*DNMT3A* ^mut^	6/9 (66.7%)	0.665	0.600 (0.114-3.153)	3/9 (33.3%)	0.700	0.583 (0.119-2.849)
*DNMT3A* ^wt^	20/26 (76.9%)			12/26 (46.2%)		
*NPM1* type A^mut^	3/7 (42.9%)	**0.055**	0.163 (0.027-0.969)	2/7 (28.6%)	**0.669**	0.462 (0.076-2.793)
*NPM1* type A^wt^	23/28 (82.1%)			13/28 (46.4%)		
*CEBPα* biallelic^mut^	5/7 (71.4%)	1.000	0.833 (0.131-5.297)	4/7 (57.1%)	0.669	2.061 (0.385-11.035)
*CEBPα* biallelic^wt^	21/28 (75.0%)			11/28 (39.3%)		
*WT1* ^mut^	6/7 (85.7%)	0.648	2.400 (0.248-23.236)	4/7 (57.1%)	0.669	2.061 (0.385-11.035)
*WT1* ^wt^	20/28 (71.4%)			11/28 (39.3%)		
*TET2* ^mut^	3/6 (50.0%)	0.156	0.261 (0.042-1.635)	3/6 (50.0%)	1.000	1.417 (0.243-8.256)
*TET2* ^wt^	23/29 (79.3%)			12/29 (41.4%)		
*IDH1/IDH2* ^mut^	4/5 (80.0%)	0.747	1.455 (0.141-15.039)	2/5 (40.0%)	1.000	0.872 (0.127-6.003)
*IDH1/IDH2* ^wt^	22/30 (73.3%)			13/30 (43.3%)		
*RUNX1* ^mut^	4/4 (100.0%)	**0.303**	Not reached	4/4 (100.0%)	**0.026**	Not reached
*RUNX1* ^wt^	21/31 (67.7%)			11/31 (35.9%)		
*NRAS* ^mut^	2/3 (66.7%)	0.758	0.667 (0.053-8.372)	2/3 (66.7%)	0.794	2.923 (0.239-35.681)
*NRAS* ^wt^	24/32 (75.0%)			13/32 (40.6%)		
*FLT3*-TKD^mut^	2/3 (66.7%)	0.758	0.667 (0.053-8.372)	2/3 (66.7%)	0.794	2.923 (0.239-35.681)
*FLT3-*TKD^wt^	24/32 (75.0%)			13/32 (40.6%)		

OR, odds ratio. The bolded text means that there are significant differences between groups.

The ORR for the 25 CDIAG induction courses in 23 refractory patients was 76.0% (19/25) (7 of 8 courses in 8 patients who had IF after 1 course of IT, 9 of 12 courses in 10 patients who had IF after 2 consecutive courses of IT, and only 3 of 5 courses in 5 patients who had IF after ≥ 3 consecutive courses of IT or relapsed ≥ twice). The CR/CRi rate for the 25 courses in 23 refractory patients was 48.0% (12/25) (4 of 8 courses in 8 patients who had IF after 1 course of IT, 7 of 12 courses in 10 patients who had IF after 2 consecutive courses of IT, and only 1 of 5 patients who had IF after ≥ 3 consecutive courses of IT or relapsed ≥ twice achieved CR/CRi by CDIAG reinduction). Among all the refractory subgroups, the best CRR of 58.3% was achieved in 12 courses of 10 patients who had IF after 2 consecutive courses of IT (**Table 3**). The 2-year OS and PFS rates for the three refractory groups were 28.6%, 60.0%, 0% and 28.6%, 54.0%, 0.0%, respectively (**Table 4**).

**Table 4:** Overall survival and progress-free survival univariate analysis.

Variable	Alive (%)	HR (95%CI)	Median OS(months)	2-year OS (%)	P-Value	HR (95%CI)	Median PFS(months)	2-year PFS (%)	P-Value
Overall	17/35(48.6)	-	11.7	38.2	-	-	11.7	37.8	-
Age									
< 51 years	13/25(52.0)	1.470(0.470-4.605)	10.1	32.1	0.545	1.586(0.525-4.796)	10.8	31.3	0.462
≥ 51 years	4/7(57.1)	0.680(0.217-2.130)	Not reached	53.6		0.630(0.209-1.906)	Not reached	53.3	
Sex									
Male	10/19(52.6)	0.642(0.213-1.98)	18.4	43.5	0.391	0.711(0.244-2.073)	11.7	41.5	0.501
Female	7/13(53.8)	1.558(0.516-4.701)	10.1	29.0		1.407(0.483-4.104)	10.8	31.1	
BM-Blast									
< 0.3	3/6(42.9)	1.550(0.359-6.698)	8.9	Not reached	0.487	1.458(0.350-6.070)	7.4	Not reached	0.549
≥ 0.3	14/26(53.8)	0.645(0.149-2.787)	13.8	39.5		0.686(0.165-2.857)	13.8	38.9	
WBC									
< 14 × 10E9/L	5/10(50.0)	1.151(0.383-3.464)	10.1	30.9	0.795	1.052(0.362-3.058)	10.8	30.9	0.925
≥ 14 × 10E9/L	12/22(54.5)	0.869(0.289-2.613)	13.8	40.5		0.951(0.327-2.765)	13.8	40.1	
HB									
< 65 g/L	3/9(33.3)	2.240(0.672-7.463)	7.9	15.2	0.112	2.067(0.639-6.684)	7.4	15.2	0.147
≥ 65 g/L	14/23(60.9)	0.447(0.134-1.488)	13.8	49.2		0.484(0.150-1.564)	13.8	47.7	
PLT									
< 40 × 10E9/L	7/13(53.8)	0.790(0.286-2.183)	18.4	34.0	0.652	0.966(0.360-2.587)	11.7	31.1	0.944
≥ 40 × 10E9/L	10/19(52.6)	1.265(0.458-3.494)	10.1	39.0		1.036(0.387-2.776)	10.1	40.9	
Previous HMA or not									
Yes	3/7(42.6)	1.885(0.477-7.449)	8.9	Not reached	0.267	1.830(0.471-7.110)	5.4	Not reached	0.285
No	14/25(51.9)	0.531(0.134-2.097)	13.8	38.9		0.546(0.141-2.123)	11.7	37.9	
Prognostic score of R/R AML^†^									
Favorable/intermediate risk	2/6(33.3)	1.750(0.458-6.687)	8.9	20.0	0.324	1.485(0.419-5.271)	7.9	20.0	0.484
Adverse risk	15/26(57.7)	0.572(0.150-2.185)	18.4	43.4		0.673(0.190-2.390)	13.8	42.4	
Relapsed/Refractory									
Relapsed	6/9(66.7)	0.601(0.198-1.825)	Not reached	51.4	0.422	0.489(0.171-1.397)	Not reached	51.4	0.250
Refractory	12/23(52.2)	1.664(0.548-5.055)	11.7	32.7		2.045(0.716-5.844)	11.7	31.5	
Relapsed/Refractory subgroup									
Early relapse	2/4(50.0)	-	10.1	33.3	**0.011**	-	10.1	33.3	**0.044**
Late relapse	4/5(80.0)		Not reached	66.7			Not reached	66.7	
IF after 1 course of IT	3/8(37.5)		13.8	28.6			13.8	28.6	
IF after 2 consecutive courses of IT	7/10(70.0)		Not reached	60.0			Not reached	54.0	
IF after ≥ 3 consecutive courses of IT or relapse ≥ twice	1/5(20.0)		7.4	0.0			5.4	0.0	
Genes Mutated									
*FLT3*-ITD^mut^	5/9(55.6)	1.014(0.322-3.195)	13.8	45.7	0.981	0.936(0.307-2.850)	13.8	48.6	0.908
*FLT3*-ITD^wt^	12/23(52.2)	0.987(0.313-3.110)	10.8	35.6		1.068(0.351-3.253)	10.8	34.4	
*DNMT3A* ^mut^	6/9(66.7)	0.516(0.175-1.519)	Not reached	62.2	0.292	0.701(0.246-1.999)	Not reached	53.3	0.531
*DNMT3A* ^wt^	11/23(47.8)	1.937(0.658-5.701)	10.1	28.7		1.427(0.500-4.071)	10.1	29.8	
*NPM1* type A^mut^	3/7(42.9)	1.765(0.460-6.773)	13.8	26.8	0.320	1.407(0.405-4.886)	13.8	26.8	0.549
*NPM1* type A^wt^	14/25(56.0)	0.567(0.148-2.174)	11.7	41.4		0.711(0.205-2.469)	11.7	40.0	
*CEBPα* biallelic^mut^	4/7(57.1)	1.081(0.296-3.948)	8.9	42.9	0.904	1.430(0.409-4.999)	7.4	35.7	0.531
*CEBPα* biallelic^wt^	13/25(52.0)	0.925(0.253-3.380)	11.7	36.5		0.699(0.200-2.444)	13.8	37.4	
*WT1* ^mut^	4/7(57.1)	1.160(0.308-4.365)	10.8	25.0	0.816	1.015(0.288-3.585)	11.7	26.7	0.981
*WT1* ^wt^	13/25(52.0)	0.862(0.229-3.243)	13.8	40.6		0.985(0.279-3.477)	13.8	39.1	
*TET2* ^mut^	3/6(50.0)	1.866(0.389-8.947)	7.4	27.8	0.319	1.989(0.492-8.045)	7.4	22.2	0.218
*TET2* ^wt^	14/26(53.8)	0.536(0.112-2.571)	13.77	39.8		0.503(0.124-2.034)	13.8	40.7	
*IDH1/IDH2* ^mut^	5/5(100.00)	0.280(0.079-0.997)	Not reached	100.0	**0.050**	0.278(0.082-0.939)	Not reached	100.0	**0.039**
*IDH1/IDH2* ^wt^	12/27(44.4)	3.566(1.003-12.680)	10.1	28.8		3.595(1.065-12.140)	10.1	28.2	
*RUNX1* ^mut^	0/4(0.00)	0.294(0.053-1.630)	7.8	0.0	**0.023**	0.283(0.495-1.622)	4.5	0.0	**0.018**
*RUNX1* ^wt^	18/31(58.1)	3.405(0.613-18.900)	18.4	46.1		3.531(0.616-20.220)	17.4	44.6	
*NRAS* ^mut^	1/3(33.3)	1.413(0.258-7.736)	10.8	0.0	0.642	1.485(0.263-8.394)	11.7	0.0	0.593
*NRAS* ^wt^	16/29(55.2)	0.708(0.129-3.877)	13.8	41.4		0.673(0.119-3.805)	13.8	40.5	
*FLT3*-TKD^mut^	1/3(33.3)	1.084(0.234-5.027)	13.8	33.3	0.915	1.022(0.230-4.555)	13.8	33.3	0.976
*FLT3-*TKD^wt^	16/29(55.2)	0.922(0.199-4.277)	11.7	38.7		0.978(0.220-4.357)	11.7	38.1	
Response									
Yes	15/25(60.0)	0.296(0.066-1.341)	18.4	46.8	**0.015**	0.358(0.087-1.469)	17.4	46.0	**0.041**
No	2/7(28.6)	3.374(0.746-15.260)	7.4	0.0		2.797(0.681-11.490)	7.4	0.0	
CR/CRi									
Yes	10/15(66.7)	0.384(0.1384-1.067)	Not reached	58.7	**0.067**	0.511(0.191-1.365)	Not reached	57.3	**0.179**
No	7/17(41.2)	2.603(0.938-7.224)	10.1	18.6		1.959(0.733-5.236)	10.1	18.7	
MRD									
≥ 10^-1^	2/6(33.3)	-	7.40	0.0	**< 0.0001**	-	7.4	0.0	**0.005**
<10^-1^ and ≥10^-3^	8/15(53.3)		18.4	43.1			17.4	40.2	
<10^-3^	7/8(87.5)		Not reached	75.0			Not reached	75.0	
SCT or not									
Yes	11/19(57.9)	0.485(0.160-1.467)	18.4	45.6	**0.150**	0.639(0.226-1.810)	17.4	44.3	**0.366**
No	6/13(46.2)	2.062(0.682-6.235)	7.9	24.2		1.564(0.553-4.427)	10.8	25.4	
SCT with a response or not									
Yes	9/15(60.0)	0.194(0.011-3.528)	Not reached	51.3	**0.017**	0.387(0.044-3.430)	17.4	48.9	**0.204**
No	2/4(50.0)	5.146(0.283-93.420)	7.4	0.0		2.584(0.292-22.910)	7.4	0.0	
SCT with CR/CRi or not									
Yes	8/12(66.7)	0.438(0.097-1.975)	Not reached	60.0	0.227	0.605(0.150-2.440)	Not reached	57.1	0.447
No	3/7(42.9)	2.282(0.506-10.290)	10.1	22.9		1.651(0.410-6.650)	10.1	22.9	
Responders underwent SCT or not									
Yes	9/15(60.0)	0.493(0.117-2.075)	Not reached	51.3	0.260	0.710(0.193-2.617)	17.4	48.9	0.579
No	6/10(60.0)	2.027(0.482-8.521)	10.8	36.0		1.408(0.382-5.186)	10.8	39.4	
CR/CRi underwent SCT or not									
Yes	8/12(66.7)	0.523(0.341-8.106)	Not reached	60.0	0.558	0.956(0.108-8.450)	Not reached	57.1	0.965
No	2/3(66.7)	1.902(0.123-29.320)	13.2	Not reached		1.049(0.118-9.294)	Not reached	66.7	

^†^Prognostic score was graded by the European Prognostic Index score in ELN. HR, hazards ratio. The bolded text means that there are significant differences between groups.

**Figure 3:**
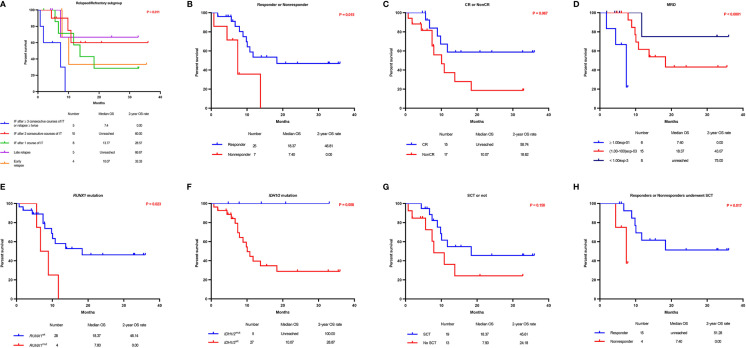
Overall survival according to prognostic characteristics and treatment allocation. Kaplan–Meier graphs illustrating the overall survival of 32 R/R AML patients with 5 different subgroups **(A)**, responders *versus* nonresponders **(B)**, patients who achieved CR *versus* those who did not **(C)**, patients according to the MRD status **(D)**, patients with *RUNX1* ^mut^ *versus RUNX1* ^wt^ **(E)**, patients with *IDH* ^mut^ *versus IDH* ^wt^ **(F)**, patients who received SCT *versus* those who did not **(G)**, and responders who underwent SCT *versus* nonresponders who underwent SCT **(H)**.

Five subgroups among the entire cohort of R/R AML patients had different OS and PFS rates (P = 0.011 and 0.044, respectively), in which patients who had IF after ≥ 3 consecutive courses of IT or relapsed ≥ twice had the worst survival rate, and patients who had late relapse achieved the best survival rate (**Table 4**, **Figure 3A**, and **Supplemental Figure 1A**). The 2-year OS and PFS rates of relapsed and refractory patients were 51.4% **vs**. 32.7% and 51.4% **vs**. 31.5% (P = 0.422 and 0.250), respectively.

The median OS and PFS times for patients who achieved a response were 18.4 and 17.4 months, respectively, while those for nonresponders were 7.4 and 7.4 months, respectively. Additionally, OS and PFS were significantly longer in responders than in nonresponders (P = 0.015 and 0.041, respectively) (**Table 4**, **Figure 3B**, and **Supplemental Figure 1B**). The 2-year OS and PFS rates for the 25 patients who achieved a response were 46.8% and 46.0%, respectively, while those for nonresponders (7 patients without a response after CDIAG) were 0.0% and 0.0%, respectively. The median OS and PFS rates for 15 patients who achieved CR were not available, while those for patients who did not were 10.1 and 10.1 months, respectively. The 2-year OS and PFS rates for patients who achieved CR were 58.7% and 57.3%, respectively, while those for 17 patients who could not achieve CR after CDIAG were 18.6% and 18.7%, respectively (P = 0.067 and 0.179, respectively) (**Table 4**, **Figure 3C**, and **Supplemental Figure 1C**).

The minimal residual disease (MRD) of flow cytometry (FCM) was analyzed in 29 patients and divided into the following three groups: 6 patients had MRD ≥ 10-1, 15 patients had MRD <10-1 and >10-3, 8 patients had MRD ≤10-3. The OS of the three groups were consistent with the clinical estimate. The lowest MRD group achieved the best survival (2-year OS rate: 75.0%), the MRD ≥ 10-1 group showed the worst OS and PFS (2-year OS rate: 0.0%), and the survival of the MRD <10-1 and >10-3 group was intermediate (2-year OS rate, 43.1 months). The survival difference among the three groups was statistically significant (OS: P < 0.0001; PFS: P = 0.005) (**Table 4**, **Figure 3D**, and **Supplemental Figure 1D**).

Among the 32 evaluable patients, all 4 with a RUNX1 gene mutation achieved CR after one course of the CDIAG regimen. However, in 28 patients with wild-type RUNX1, the response rate for 31 CDIAG induction courses was 67.7% (21/31), and the CR rate was 35.9% (11/31). The CRR in the RUNX1mut group was significantly higher than that in the RUNX1wt group (P = 0.026) (**Table 3**). The presence of the RUNX1 mutations was associated with a short median OS (7.8 *vs*. 18.4 months; P = 0.023) and PFS (4.5 *vs*. 17.4 months; P = 0.018) times, with a 2-year OS rate of 0.0% *vs*. 46.1% and a 2-year PFS rate of 0.0% *vs*. 44.6% (**Table 4**, **Figure 3E**, and **Supplemental Figure 1E**).

No significant difference was found in the response rate between five IDHmut (including IDH1 and IDH2) patients and 22 IDHwt patients (80.0% *vs*. 73.3%; P = 0.747). All five IDHmut patients were still alive. The survivals of these two groups were obviously different (2-year OS rate: 100.0% *vs*. 28.9%, P = 0.050; 2-year PFS rate, 100.0% *vs*. 28.2%, P = 0.039) (**Table 4**, **Figure 3F**, and **Supplemental Figure 1F**).

The ORR for 10 courses in 9 FLT3-ITDmut patients after the CDIAG regimen reached 70.0% (7/10) and that for 25 courses in 23 FLT3-ITDwt patients reached 76.0% (19/25). The CRR for 10 courses in 9 FLT3-ITDmut patients reached 30.0% (3/10) and that for 25 courses in 23 FLT3-ITDwt patients reached 48.0% (12/25). No difference was found in the ORR or CRR between FLT3-ITDmut and FLT3-ITDwt patients (P = 0.694 and 0.458, respectively), but FLT3-ITDwt patients showed a shorter median OS time (10.8 *vs*. 13.8 months, P = 0.981) and PFS time (10.77 *vs*. 13.77 months, P = 0.908) (**Table 4**). Additionally, no significant correlation was found between mutations in other genes (CEBPα, DNMT3A, NPM1, TET2, WT1, NRAS, FLT3-TKD) and the remission or survival rate.

Among the entire cohort, nineteen of the 32 eligible patients (59.4%) successfully bridged to SCT after CDIAG treatment. The 2-year OS and PFS rates of the 19 patients who had undergone SCT were 45.6% and 44.3%, respectively, and the rates of the 13 patients who did not undergo SCT were 24.2% and 25.4%, respectively. No significant difference was found in the OS or PFS between these groups (P = 0.150 and 0.366, respectively) (**Table 4**, **Figure 3G**, and **Supplemental Figure 1G**).

Among the 19 patients who had undergone SCT, the 2-year OS rate of 15 responders was significantly higher than that of 4 nonresponders (51.3% *vs*. 0.0%; P = 0.017), but no difference was found in the 2-year PFS rate (48.9% *vs*. 0.0%; P = 0.204) (**Table 4**, **Figure 3H**, and **Supplemental Figure 1H**). For the 25 responders, the 2-year OS and PFS rates of the 15 responders who had undergone SCT were not significantly different from those of the 10 responders who had not undergo SCT (51.3% *vs*. 36.0%, P = 0.260; 48.9% *vs*. 39.4%, P = 0.579).

Of the 10 patients who achieved a response after CDIAG but did not receive SCT, four (including one who achieved CR) died from PD, two were lost to follow-up with a PD status, two (including one who achieved CR) were alive with a PD status, and only two (including one who achieved CR) were alive with a remission status under chemotherapy at the time of analysis.

At the time of analysis, five of 10 patients who received SCT from haploidentical donors survived and achieved CR; 1 died because of TRM, 3 died from relapse, and one was alive after relapse. One patient who received SCT from a sibling donor was lost to follow-up, and 1 patient who received a transplant from an unrelated donor remained alive and achieved CR. No early TRM (within 60 days of SCT) occurred in the 19 patients who had undergone SCT after the CDIAG regimen.

No difference was found in the ORR (71.4%, 5/7 *vs*. 80.0%, 20/25; P = 0.632), CRR (42.9%, 3/7 *vs*. 48.0%, 12/25; P = 0.576), median OS time (8.9 *vs*. 13.8 months, P = 0.267) or median PFS time (5.4 *vs*. 11.7 months, P = 0.285) between patients who had been treated with or without HMA (primary decitabine). Additionally, the 2-year OS and PFS rates were not significantly different between the groups (not reached *vs*. 38.9% and not reached *vs*. 37.9% (**Table 4**).

No significant difference was found in the ORR and CRR between groups with different prognosis risks: the ORR and CRR for 7 courses in 6 patients with a favorable or intermediate risk were 71.4% (5/7) and 28.6% (2/7), respectively, while those for 28 courses in 26 patients with an adverse risk were 75.0% (21/28) and 46.4% (13/28), respectively (P = 1.000 and 0.672, respectively); the 2-year OS rates were 20.0% *vs*. 43.4%, and the 2-year PFS rates were 20.0% *vs*. 42.4%, respectively, comparing the two groups (P = 0.324 and 0.484, respectively) (**Table 4** and **Supplemental Table 2**).

Age, sex, BM blasts, the white blood cell (WBC) count, the hemoglobin count and the platelet (PLT) count did not affect the response rates.

### Safety

Thirty-five patients received 38 courses of IT. Thus, all the toxicological evaluations were performed during these 38 courses.

For hematological adverse events (AEs), the median time for neutropenia was 18.4 (0-77) days, and G-CSF injections were administered in 34 of 38 courses because the neutrophil count was less than 1.0×10exp9/L. All the patients received red blood cell (RBC) transfusions at an average of 6 Units (1200 mL) because the hemoglobin levels were below 60 g/L. Additionally, all the patients required platelet transfusions at an average of 7.3 units per course because the platelet counts were below 10×10exp9/L.

The nonhematological AEs are summarized in **Supplemental Table 3**. Two (5.3%) patients died of AEs that were deemed treatment-related (both because of severe deterioration of liver and kidney function after the infective shock). Overall, the treatment was well tolerated, although most side effects were grade 3. The reason for this high rate of grade 3 AEs was that, in most cases, inflammation required intravenous treatment and blood transfusion in the hypoimmune state.

## Discussion

The outcome of R/R AML remains poor, and treatment options are very limited. Exploring an effective and well-tolerated combination therapy is urgent. In the preclinical studies, chidamide and decitabine, two epigenetic modifiers, revealed a significant synergistic effect in both AML cell lines and primary R/R AML cells. In this phase II prospective multicenter trial, of the 32 evaluable patients treated with the CDIAG regimen, the ORR was 74.3% and CR/CRi rate was 42.9%, with a median OS of 11.7 months and a 2-year OS rate of 38.2%. Patients who achieved a response or MRD levels below 10^-3^ have a significantly better OS and PFS than those without. The clinical results were encouraging because many poor-risk individuals were enrolled and 81% of the patients had adverse cytogenetics.

SCT was plausibly the best salvage treatment option for R/R AML until the development of effective and available novel drugs (15). SCT for AML yields good results when administered to patients in a CR status (16). In a previously published prospective study, sixty-seven percent of remitters received allo-transplantation in CR2, providing a superior survival rate than no stem cell transplantation (5-year OS rate: 42% *vs*. 16%) (17). In our study, 19 patients bridged to SCT after the CDIAG regimen. Their 2-year overall survival rate was higher than that of the non-SCT group (45.6% *vs*. 24.2%; P=0.150). The results were consistent with our expectations, suggesting that the CDIAG protocol could reduce the leukemia burden before transplantation and provide a bridge for subsequent transplantation. Responders after CDIAG should receive transplantation as soon as possible.

Among our entire cohort of refractory and relapsed patients, those with PIF after ≥ 3 consecutive courses of IT or who relapsed ≥ twice had the worst OS and PFS. The 2-year survival rate of these patients was 0.0%. The patients with a late relapse had the best survival rate of 66.7%. Importantly, the survival rate of the refractory patients receiving one course of IT was worse than that of patients who received two consecutive courses of IT because of the high proportion (4/8) of *FLT3*-ITD mutations in the former group. Most studies thus far have suggested no difference in the response rate with or without previous HMA exposure (18). Although no significantly difference, patients who had received HMA therapy had a shorter OS time than those who had not. The median OS time was 8.90 months for previous HMA exposure vs. 13.8 months for no previous HMA exposure (*P* = 0.267). (**Table 4**). The possible mechanism underlying the shorter tendency in the survival times of such patients could be due to the drug resistance property after screening by HMA drugs.

Importantly, the response rate was improved in patients with *RUNX1* mutations (100%; 4 of 4 patients), but the increased sensitivity could not compensate for the poor prognosis associated with *RUNX1* mutation (19). The 2-year OS and PFS rates for 28 *RUNX1* ^wt^ patients (courses=31) and 4 *RUNX1* ^mut^ patients were 46.1% vs. 0% and 44.6% *vs*. 0.0%, respectively (*P* = 0.023 and 0.018, respectively). *RUNX1* is an important regulator of myeloid differentiation and effective hematopoiesis (20). HDAC1 and 3 bind to *RUNX1* and regulate the transcription activity of *RUNX1 (* 21). Whether chidamide binds competitively to HDAC1 and 3 against *RUNX1* and plays a role in CDIAG IT deserves further exploration. Interestingly, even the presence of *IDH* mutation did not affect the CR rate but achieved better OS and PFS. Although several studies have investigated the incidence and prognosis of *IDH* mutations in patients with AML, the significance of *IDH* mutations on AML outcome has been unclear (22). Better survival might benefit from the impact of *IDH* on histone modifications and DNA methylation (23, 24). As mentioned above, no difference was found in the response or survival rate between *FLT3*-ITD^mut^ and *FLT3*-ITD^wt^ patients, but *FLT3*-ITD^mut^ patients had worse outcomes. Recently, Hu et al. revealed a novel resistance pathway involving *FLT3*-ITD^mut^: in AML cells, *FLT3*-ITD^mut^ upregulates HDAC8, thereby promoting the persistence of *FLT3*-ITD^mut^ AML cells even in the presence of an *FLT3* inhibitor (25). This view confirms our findings. *FLT3*-ITD^mut^ patients achieved a poor response, and 4 of 7 responders with *FLT3*-ITD^mut^ ultimately achieved PD with poor outcomes, likely because of the ineffectiveness of chidamide for selectively inhibiting HDAC1, 2, 3 and 10 instead of HDAC8.

Despite the clinical activity of chidamide combination therapy in R/R AML patients, toxicity is still commonly observed in this cohort. The degree of cytopenia and resulting complications reported in our study are not higher than those reported in treatment-naïve patients or other R/R populations, although the rates and degrees of baseline cytopenia were higher (26). We found that infections of grade 3 or higher were observed in nearly half of the cohort (18 courses), and 2 of the 18 courses developed infectious shock. Three patients died within 4 weeks after treatment, 2 of the 3 patients developed severe infection and shock, and one patient persistently maintained no response and died after receiving chemotherapy. Even with these toxicities, in our study, the median OS and PFS times were 11.7 and 11.7 months, respectively, and the 2-year OS and PFS rates were 38.2% and 37.8%, respectively, which are equivalent or superior to those of conventional salvage therapy (27).

## Conclusion

The CDIAG regimen was well tolerated and associated with a higher clinical response rate than expected in the context of salvage therapy for R/R AML. The regimen delays disease progression and reduces the leukemia burden before transplantation, providing eligible patients with the chance of proceeding to allo-SCT. Our results show that epigenetic agents combining cytotoxic agents may represent a promising direction for patients with R/R AML. Further evaluations in larger population are needed to seek biological indicators benefiting from this regimen.

## Data Availability Statement

The original contributions presented in the study are included in the article/**Supplementary Material**. Further inquiries can be directed to the corresponding authors.

## Ethics Statement

The studies involving human participants were reviewed and approved by the Ethics Committee of the First Affiliated Hospital of Soochow University. The patients/participants provided their written informed consent to participate in this study.

## Author Contributions

JY contributed to data curation, formal analysis, visualization, and writing-original draft. C-LW contributed to writing-original draft and visualization. LZ contributed to data curation. HZ contributed to methodology and investigation. LB contributed to methodology and investigation. H-XZ contributed to investigation and resources. M-ZX contributed to investigation and resources. L-YC contributed to investigation and resources. C-SQ contributed to investigation and resources. H-YQ contributed to methodology and validation. S-NC contributed to methodology and validation. X-WT contributed to investigation and resources. D-PW contributed to conceptualization, supervision, and writing-review and editing. Z-YM contributed to methodology and investigation. A-NS contributed to conceptualization, funding acquisition, supervision, and writing-review. S-LX contributed to conceptualization, funding acquisition, supervision, resources, and writing-review. All authors contributed to the article and approved the submitted version.

## Funding

This work was supported by grants from the National Natural Science Foundation of China (Grant No. 81970138), Translational Research Grant of NCRCH (Grant No. 2020ZKMB05), Jiangsu Province “333” project, Jiangsu Province Medical Youth Talent Program (Grant No. QNRC2016719), a C class sponsored project from Jiangsu Provincial Six Talent Peaks (Grant No. 2016-WSN-123) and Gusu Key Medical Talent Program (Grant No. GSWS2019007).

## Conflict of Interest

The authors declare that the research was conducted in the absence of any commercial or financial relationships that could be construed as a potential conflict of interest.

## Publisher’s Note

All claims expressed in this article are solely those of the authors and do not necessarily represent those of their affiliated organizations, or those of the publisher, the editors and the reviewers. Any product that may be evaluated in this article, or claim that may be made by its manufacturer, is not guaranteed or endorsed by the publisher.
