# Supplementary material for: A Phase II Trial of the Double Epigenetic Priming Regimen Including Chidamide and Decitabine for Relapsed/Refractory Acute Myeloid Leukemia
Source: Front Oncol. 2021 Sep 3;11:726926. doi: 10.3389/fonc.2021.726926 (PMC8446637; doi:10.3389/fonc.2021.726926)
Supplement: Supplementary file 1 [file DataSheet_1.docx]

Supplementary Material

1. **Supplementary Tables**

**Table S1. Study eligibility** **criteria**

| Inclusion criteria |  |
| --- | --- |
|  | Adults aged ≥ 18 and ≤ 70 years |
|  | Patients diagnosed with AML according to the 2016 WHO myeloid malignant disease diagnosis standard |
|  | Patients with AML must meet one of the following criteria, A or B:  A: Refractory disease was defined as follows: (1) failure to attain CR following exposure to at least 2 courses of standard or intensive induction therapy; or (2) failure to achieve CR and a bone marrow leukemia cell decline index (BMCDI) ≤ 50% after 1 course of standard or intensive induction therapy; or (3) relapse at least twice after CR.  B: Relapsed AML disease was defined as follows: (1) reappearance of leukemic blasts in the peripheral blood after CR; or (2) detection of more than 5% blasts in the BM not attributable to another cause (e.g., BM regeneration after consolidation therapy); or (3) extramedullary relapse. |
|  | ECOG performance status score less than 3 |
|  | Expected survival time ˃ 3 months |
|  | Patients without serious heart, lung, liver, or kidney disease |
|  | Ability to understand and voluntarily provide informed consent |
| Exclusion criteria |  |
|  | Patients who are allergic to the study drug or drugs with similar chemical structures |
|  | Pregnant or lactating women, and women of childbearing age who do not want to practice effective methods of contraception |
|  | Active infection |
|  | Active bleeding |
|  | Patients with new thrombosis, embolism, cerebral hemorrhage, or other diseases or a medical history within one year before enrollment |
|  | Patients with mental disorders or other conditions whereby informed consent cannot be obtained and where the requirements of the study treatment and procedures cannot be met |
|  | Liver function abnormalities (total bilirubin > 1.5 times the upper limit of the normal range, ALT/AST > 2.5 times the upper limit of the normal range or patients with liver involvement whose ALT/AST > 1.5 times the upper limit of the normal range), or renal anomalies (serum creatinine > 1.5 times the upper limit of the normal value) |
|  | Patients with a history of clinically significant QTc interval prolongation (male > 450 ms; female > 470 ms), ventricular heart tachycardia and atrial fibrillation, II-degree heart block, myocardial infarction attack within one year before enrollment, and congestive heart failure, and patients with coronary heart disease who have clinical symptoms and requiring drug treatment |
|  | Surgery on the main organs within the past six weeks |
|  | Drug abuse or long-term alcohol abuse that would affect the evaluation results |
|  | Patients who have received organ transplants (excepting bone marrow transplantation) |
|  | Patients not suitable for the study according to the investigator's assessment |

**Table S2: Schedule for the CDIAG regimen**

| Day | 1 | 2 | 3 | 4 | 5 | 6 | 7 | 8 | 9 | 10 | 11 | 12 | 13 | 14 | 15 | 16 | 17 |
| --- | --- | --- | --- | --- | --- | --- | --- | --- | --- | --- | --- | --- | --- | --- | --- | --- | --- |
| Chidamide  30 mg | ★ |  |  | ★ |  |  |  | ★ |  |  | ★ |  |  |  |  |  |  |
| Decitabine  20 mg/m^2^ | ◎ | ◎ | ◎ | ◎ | ◎ |  |  |  |  |  |  |  |  |  |  |  |  |
| Idarubicin  5 mg q12h |  |  |  | ◆ |  | ◆ |  | ◆ |  | ◆ |  | ◆ |  | ◆ |  |  |  |
| Ara-C  10 mg/m^2^ |  |  |  | △ | △ | △ | △ | △ | △ | △ | △ | △ | △ | △ | △ | △ | △ |
| G-CSF  200 μg/m^2^ |  |  | ↓ | ↓ | ↓ | ↓ | ↓ | ↓ | ↓ | ↓ | ↓ | ↓ | ↓ | ↓ | ↓ | ↓ | ↓ |

**Abbreviations:** Ara-C: cytarabine; G-CSF: granulocyte-colony stimulating factor; CDIAG regimen: chidamide (30 mg orally twice every week for 2 weeks on days 1, 4, 8, and 11), decitabine [20 mg/m^2^ intravenously daily for 5 days (d1-d5)], and the IAG regimen [cytarabine (10 mg/m^2^ subcutaneously every 12 hrs. on days 4-17), idarubicin (5 mg intravenously every other day on days 4, 6, 8, 10, 12, and 14), and concurrent G-CSF (200 μg/m^2^/day subcutaneously daily)].

**Table S3: Subsequent treatment of patients who did not undergo SCT**

| **No.** | **Therapy regimen** | **Survival status** |
| --- | --- | --- |
| 1 | Untreated | Alive |
| 2 | HA | Deceased |
| 3 | Microtransplantation | Deceased |
| 4 | HAAG | Deceased |
| 5 | Untreated | Deceased |
| 6 | Untreated | Deceased |
| 7 | [Sorafenib](https://www.kangbixing.com/news/59656.html) | Lost to follow-up |
| 8 | CAG, decitabine+HA | Lost to follow-up |
| 9 | Untreated | Lost to follow-up |
| 10 | Decitabine+HAAG | Alive |
| 11 | Untreated | Deceased |
| 12 | MA, MA | Deceased |
| 13 | Untreated | Deceased |
| 14 | CLL1 CAR-T | Deceased |
| 15 | Untreated | Alive |
| 16 | CD38 CAR-T | Alive |

**Abbreviations:** HA: homoharringtonine combined with Ara-C; HAAG: homoharringtonine combined with Ara-C, aclarubicin and G-CSF; CAG: Ara-C combined with aclarubicin and G-CSF; MA: mitoxantrone combined with Ara-C; CLL1: C-type lectin-like molecule-1; CAR-T: chimeric antigen receptor T-cell immunotherapy.

**Table S4:** **Definitions of events and end points**

| **Events and end points** | **Abbreviation** | **Definition** |
| --- | --- | --- |
| Primary refractory |  | Failure to attain CR following exposure to at least 2 courses of induction therapy, or failure to attain CR and a bone marrow leukemia cell decline index (BMCDI) ≤ 50% after 1 course of induction therapy, or relapse at least twice after CR. |
| Relapse |  | Reappearance of leukemic blasts in the peripheral blood, or the finding of more than 5% blasts in the bone marrow, or extramedullary relapse |
| Early relapse | ER | Relapse ≤ 6 months after CR1 |
| Late relapse | LR | Relapse > 6 months after CR1 |
| Early death |  | Death within 28 days from the start of treatment |
| Complete response | CR | Normal hematopoiesis in the bone marrow, bone marrow blasts < 5%; absence of circulating blasts and blasts with Auer rods; absence of extramedullary disease; ANC ≥ 1.0×10^9^/L (1000/μL); and platelet count ≥ 100×10^9^/L (100 000/μL) |
| CR with incomplete blood  count recovery | CRi | All CR criteria except for residual neutropenia (< 1.0×10^9^/L [1000/μL]) or thrombocytopenia (< 100×10^9^/L [100 000/μL]) |
| Morphologic leukemia-free  state | MLFS | Bone marrow blasts < 5%; absence of blasts with Auer rods;  absence of extramedullary disease; no requirement for hematologic recovery |
| Partial remission | PR | All hematologic criteria of CR; a decrease in the BM blast percentage from 5% to 25%; and a decrease in the pretreatment BM blast percentage by at least 50% |
| Stable disease | SD | Absence of CR, CRi, PR, MLFS; and criteria for PD  not met |
| Progressive disease | PD | Evidence for an increase in the BM blast percentage and/or an increase in absolute blast counts in the blood: > 50% increase in marrow blasts over baseline, or > 50% increase in peripheral blasts (WBC × % blasts) to > 25×10^9^/L (> 25 000/μL), or new extramedullary disease |
| Overall survival | OS | Measured from the onset of CDIAG treatment to the date of the last follow-up or death |
| Progression-free survival | PFS | Measured from the date of entry into the trial until the date of disease progression at any site, including distant metastasis or second primary tumors, or death |
| Relapse-free survival | RFS | Measured from the date of attaining CR1 until the first relapse, death, or the final follow-up day |

**Abbreviations:** CR1, first complete remission.

**Table S5:** **Nonhematological adverse events for 35 patients (38 courses)**

| **Factor** | **No. of courses (%)** | **Grade 3/4 AEs, No. (%)** |
| --- | --- | --- |
| Infection | 27 (71.1) | 11 (28.9) |
| Neutropenia  (except sepsis) | 20 (52.6) | 4 (10.5) |
| Sepsis | 7 (18.4) | 7 (18.4) |
| Pneumonia | 14 (36.8) | 3 (7.9) |
| Skin and soft tissue infection | 4 (10.5) | 4 (10.5) |
| Others | 6 (15.8) | 3 (7.9) |
| Nausea/vomiting | 24 (63.2) | 1 (2.6) |
| Stomatitis | 6 (15.8) | 2 (5.3) |
| Diarrhea | 11 (28.9) | 1 (2.6) |
| Anorexia | 8 (21.1) | 0.0 |
| Fatigue | 9 (23.7) | 0.0 |
| Hepatobiliary | 8 (21.1) | 1 (2.6) |
| Aminotransferase elevation | 7 (18.4) | 1 (2.6) |
| Bilirubin elevation | 1 (2.6) | 0.0 |
| Renal-Cr elevation | 1 (2.6) | 1 (2.6) |
| Others | 7 (18.4) | 0.0 |

**Abbreviations:** Cr: creatinine.

**Table S6: Characteristics of the 32 evaluable patients**

| **Characteristic** | **Value** |
| --- | --- |
| Relapsed/refractory | 9/23 |
| Male/female, No. | 19/13 |
| Age, median (range), y | 39 (18-68) |
| WBC count, median (range), ×10^9^/L | 26.7 (1.0-299.0) |
| Hemoglobin level, median (range), g/L | 77 (48-127) |
| Platelet count, median (range), ×10^9^/L | 52 (12-376) |
| BM blasts, median (range), % | 61.5 (10.0-97.5) |
| WHO classification, No. (%) |  |
| AML, NOS | 26 (81.3) |
| AML with MRC | 4 (12.5) |
| AML with t(8; 21) | 1 (3.1) |
| AML with inv(16) | 1 (3.1) |
| Prognosis risk for R/R AML, No. (%) |  |
| Favorable | 2 (6.3) |
| Intermediate | 4 (12.5) |
| Poor | 26 (81.3) |
| Prior HMA exposure (before the CDIAG regimen), No. (%) |  |
| No | 25 (78.1) |
| Yes | 7 (21.9) |
| Subgroup classification of R/R AML, No. (%) |  |
| Early relapse | 4 (12.5) |
| Late relapse | 5 (15.6) |
| IF after 1 course of IT | 8 (25.0) |
| IF after 2 consecutive courses of IT | 10 (31.3) |
| IF after ≥ 3 consecutive courses of IT | 3 (9.4) |
| Relapse ≥ twice | 2 (6.3) |
| Therapy after regimen, No. (%) |  |
| SCT | 19 (59.4) |
| Others | 13 (40.6) |
| Genes mutated, No. (%) |  |
| *FLT3*-ITD mutated | 9 (28.1) |
| *DNMT3A* mutated | 9 (28.1) |
| *NPM1* type A mutated | 7 (21.9) |
| *CEBPα* biallelic mutated | 7 (21.9) |
| *WT1* mutated | 7 (21.9) |
| *TET2* mutated | 6 (18.8) |
| *IDH1*/*IDH2* mutated | 5 (15.6) |
| *RUNX1* mutated | 4 (12.5) |
| *NRAS* mutated | 3 (9.4) |
| *FLT3*-TKD mutated | 3 (9.4) |
| *U2AF1* mutated | 2 (6.3) |
| *TP53* mutated | 1 (3.1) |

**Abbreviations:** R/R AML: relapsed/refractory AML; AML, NOS: AML, not otherwise specified; AML with MRC: AML with myelodysplasia-related changes; HMA: hypomethylating agent; SCT: stem cell transplantation; WBC: white blood cell; *FLT3*: FMS-like tyrosine kinase 3, *FLT3*-ITD: *FLT3*-internal tandem duplication; *DNMT3A*: DNA-methyltransferase 3A; *NPM1*: nucleophosmin 1; *CEBPα*: CCAAT/enhancer binding protein alpha; *WT1*: Wilms’ tumor 1; *IDH1*: isocitrate dehydrogenase 1; *IDH2*: isocitrate dehydrogenase 2; *TET2*: Tet methylcytosine dioxygenase 2; *RUNX1*: runt-related transcription factor 1; *NRAS*: neuroblastoma RAS viral oncogene homolog; *TP53*: tumor protein 53; *FLT3*-TKD: *FLT3*-tyrosine kinase domain; *U2AF1*: U2 small nuclear RNA auxiliary factor 1.

**Table S7: Outcomes according to the prognostic index (****European Prognostic Index) in ELN**

| **Group** | **No. (%)** | **Median score** | **5-year survival rate** |
| --- | --- | --- | --- |
| Favorable | 2 (6.3) | 5 | 46% |
| Intermediate | 4 (12.5) | 8 | 18% |
| Poor | 26 (81.3) | 11 | 4% |

- 1. **Supplementary Figures**


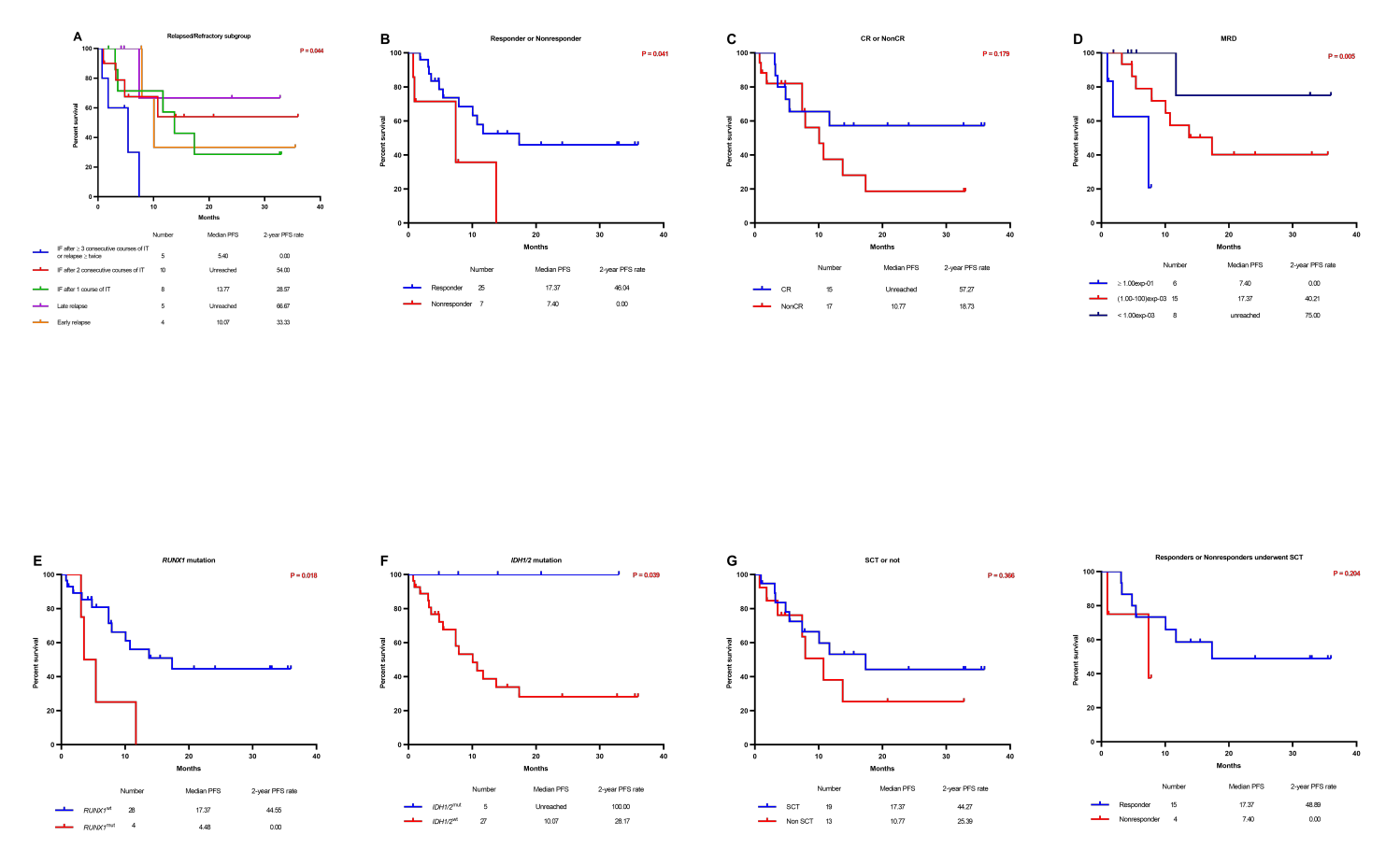


**Supplementary Figure 1. Progression-free survival according to prognostic characteristics and treatment allocation.**

Kaplan–Meier graphs illustrating the progression-free survival of 32 R/R AML patients with 5 different subgroups (A), responders versus nonresponders (B), patients who achieved CR versus those who did not (C), patients according to the MRD status (D), patients with *RUNX1*^mut^ versus *RUNX1*^wt^ (E), patients with *IDH*^mu^t versus *IDH*^wt^ (F), patients who received SCT versus those who did not (G), and responders who underwent SCT versus nonresponders who underwent SCT (H).
